# Prevalence of methicillin-resistant *Staphylococcus haemolyticus* in companion animals: a cross-sectional study

**DOI:** 10.1186/s12941-014-0056-y

**Published:** 2014-11-28

**Authors:** Modestas Ruzauskas, Rita Siugzdiniene, Irena Klimiene, Marius Virgailis, Raimundas Mockeliunas, Lina Vaskeviciute, Dainius Zienius

**Affiliations:** Microbiology and Virology Institute, Lithuanian University of Health Sciences, Mickeviciaus g. 9, Kaunas, LT44307 Lithuania

**Keywords:** *Staphylococcus haemolyticus*, Methicillin-resistance, Kennels, Antimicrobial resistance, Companion animals

## Abstract

**Background:**

Among coagulase-negative staphylococci, *Staphylococcus haemolyticus* is the second most frequently isolated species from human blood cultures and has the highest level of antimicrobial resistance. This species has zoonotic character and is prevalent both in humans and animals. Recent studies have indicated that methicillin-resistant *S. haemolyticus* (MRSH) is one of the most frequent isolated *Staphylococcus* species among neonates in intensive care units. The aim of this study was to determine the presence of MRSH in different groups of companion animals and to characterize isolates according their antimicrobial resistance.

**Methods:**

Samples (n = 754) were collected from healthy and diseased dogs and cats, female dogs in pure-breed kennels, healthy horses, and kennel owners. Classical microbiological tests along with molecular testing including PCR and 16S rRNA sequencing were performed to identify MRSH. Clonality of the isolates was assessed by Pulsed Field Gel Electrophoresis using the *Sma*I restriction enzyme. Antimicrobial susceptibility testing was performed using the broth micro-dilution method. Detection of genes encoding antimicrobial resistance was performed by PCR. Statistical analysis was performed using the R Project of Statistical Computing, “R 1.8.1” package.

**Results:**

From a total of 754 samples tested, 12 MRSH isolates were obtained. No MRSH were found in horses and cats. Eleven isolates were obtained from dogs and one from a kennel owner. Ten of the dog isolates were detected in pure-breed kennels. The isolates demonstrated the same clonality only within separate kennels.

The most frequent resistances of MRSH isolates was demonstrated to benzylpenicillin (91.7%), erythromycin (91.7%), gentamicin (75.0%), tetracycline (66.7%), fluoroquinolones (41.7%) and co-trimoxazole (41.7%). One isolate was resistant to streptogramins. All isolates were susceptible to daptomycin, rifampin, linezolid and vancomycin. The clone isolated from the kennel owner and one of the dogs was resistant to beta-lactams, macrolides, gentamicin and tetracycline.

**Conclusions:**

Pure-breed kennels keeping 6 or more females were determined to be a risk factor for the presence of MRSH strains. MRSH isolated from companion animals were frequently resistant to some classes of critically important antimicrobials, although they remain susceptible to antibiotics used exclusively in human medicine.

## Background

The ongoing change in the relationship between humans and companion animals is hallmarked by increasing intensive care provided to companion animals in veterinary medicine, resulting in growing numbers of high-risk animal patients [1]. Due to the zoonotic potential of resistant bacteria and the close contact of pets with their owners, investigations on the presumptive transmission and infection routes of different bacterial species should be performed. The bacteria genus *Staphylococcus* is one of the most prevalent in animals and humans. Methicillin-resistant staphylococci are recognized as one of the most important threats for human and animal health [2,3]. In spite of methicillin-resistant *S. aureus* (MRSA) being recognized as the most important *Staphylococcus* species in human patients, the other species also cause severe infections [4,5]. These species mostly include coagulase-negative staphylococci (CoNS) such as *S. epidermidis*, *S. saprophyticus,* and many other species within this group [6].

Among CoNS, *Staphylococcus haemolyticus* is the second most frequently isolated species from human blood cultures, and has the highest level of antimicrobial resistance [6-8]. This species may also cause septicemia, peritonitis, otitis, and urinary tract infections [6]. Recent studies reveled that methicillin-resistant *S. haemolyticus* (MRSH) was one of the most frequently isolated *Staphylococcus* species among neonates in intensive care units [9,10]. Data about the prevalence of this species in companion animals are still scarce. Research from the Netherlands has found 4 isolates of MRSH from 11 multidrug-resistant staphylococci isolated from dogs and cats [11]. In Denmark, an MRSH clone was isolated from horses, staff members, and environmental sites [12]. Another methicillin-resistant clone was obtained from cat wounds and animal cages in Norway [13].

In clinical laboratory practice, it is often uncommon to identify CoNS up to the species level for different reasons, such as high species variety, unclear taxonomic position, and unreliable identification results using biochemical tests [6,14]. Moreover, there are no special treatment strategies suggested for infections caused by different CoNS species. Therefore, there is a lack of data about the prevalence of certain CoNS species in both humans and animals. Such data are important, as different *Staphylococcus* species might have different pathogenicity mechanisms, pathogenesis, or peculiar transmission aspects. It is particularly important to identify methicillin-resistant CoNS species in companion animals for better understanding of microbiome and disease ecology, as well as for the selection of more appropriate control options in case they are needed. The aim of this study was to determine the presence of MRSH as a possible zoonotic agent in companion animals and to characterize the isolates according their antimicrobial resistance.

## Materials and methods

### Animals and sampling

From 2012 to 2014, samples were collected from 450 dogs, 50 cats, and 250 horses in Lithuania. The samples included diseased (300 dogs, 50 cats, and 50 horses) and healthy animals (200 horses and 50 dogs). Samples from the diseased pets (skin and wound infections, otitis, keratitis, infections of the respiratory and gastro-intestinal tracts) were taken directly at small animal clinics. Samples from the diseased horses (wounds, respiratory and gastro-intestinal tract infections) were taken at stables or directly on pastures. Sampling of diseased animals was performed from the sites of infection, whereas nasal (30 from dogs and 150 from horses) and groin (20 from dogs and 50 from horses) samples were taken from healthy animals. One hundred vaginal samples were taken from adult female dogs suffering from reproductive organ infections or in cases of other reproductive disorders (infertility, *partus praematurus*, abortion) directly at kennels. Samples were collected by veterinary surgeons using sterile Amies media swabs (Liofilchem, Italy) or other necessary instruments under aseptic conditions from the affected organs or presumptive places of infection. Samples were delivered to the laboratory within the same day. Anamneses data were analysed in case of MRSH detection. Four owners of the kennels voluntarily presented their own nasal samples using sterile cotton swabs. Ethics approval was obtained from Bioethics Centre of Lithuanian University of Health Sciences. Informed consent was obtained from each volunteer.

### Bacteriological analysis

Clinical material was inoculated onto 5% Sheep Blood Agar, Mannitol Salt Agar (Liofilchem, Italy) supplemented with 4 mg/L cefoxitin (Sigma-Aldrich) and Brilliance MRSA 2 Agar (Oxoid, Thermo Fisher, UK). Presumptive identification of *Staphyloccus* genus was based on the growth and morphology characteristics, catalase production, gram-staining and susceptibility to furazolidone. Species identification was performed only for the isolates that grew on Mannitol Salt Agar supplemented with 4 mg/L cefoxitin and/or and Brilliance MRSA 2 Agar. Single colonies were taken from the agar surface and re-cultivated on the Mannitol Salt Agar supplemented with cefoxitin and Brilliance MRSA 2 Agar with the aim to obtain pure cultures. Presumptive species identification was based on pigment and coagulase production, presence of protein A and clumping factor as well as on biochemical properties detected by using RapID Staph Plus (Thermo Scientific) identification system. In uncertain identification cases Matrix-Assisted Laser Desorption/Ionization Time-of-Flight (MALDI-TOF) analysis (VITEK® MS, Biomérieux, France) was used as described previously [15].

### Molecular testing

DNA material for molecular testing was obtained after bacterial lysis according to the extraction protocol prepared by the Community Reference Laboratory for Antimicrobial Resistance with slight modifications [16]. Briefly, a loopful of colonies were taken from the surface of Mueller Hinton Agar and transferred to phosphate buffered saline (pH 7.3). The content was centrifuged for 5 min. Then the supernatant was discarded and the pellet was re-suspended in Tris-EDTA (TE) buffer. The suspension was heated using a thermomixer at 100°C degrees for 10 minutes. Boiled suspension was transferred directly on ice and diluted by 1:10 in TE.

Genus specific 16S rRNA gene was investigated by PCR using *Staphylococcus* genus specific oligonucleotide primers (Table 1). MRSH confirmation was obtained through detection of the *mecA, mecC* and *S. haemolyticus* species-specific *nuc* gene (Table 1). Species verification additionally was performed using 16S rRNA sequencing. The universal primers 27 F and 515R were used as described previously [17]. Sequences were analysed using Molecular Evolutionary Genetic Analysis software (MEGA, version 6). Basic local alignment search tool (BLAST) was used for comparison of obtained sequences with sequences presented in the database of National Centre of Biotechnology Information.

**Table 1:** **Oligonucleotide primers used in this study**

**Primer name**	**Sequence (5' - 3')**	**Size, bp and T(°C)**	**Target gene**	**Source**
mecA1	GGGATCATAGCGTCATTATTC	527 (61)	*mecA*	[16]
mecA2	AACGATTGTGACACGATAGCC			
mecC1	GCTCCTAATGCTAATGCA	204 (50)	*mecC*	[18]
mecC2	TAAGCAATAATGACTACC			
16S1	GTGCCAGCAGCCGCGGTAA	886 (61)	*16S* staph	[16]
16S2	AGACCCGGGAACGTATTCAC			
hae1	TAGTGGTAGGCGTATTAGCC	434 (58)	*nuc S. haemolyticus*	[19]
hae2	ACGATATTTGCCATTCGGTG			
blaZ1	CAGTTCACATGCCAAAGAG	772 (50)	*blaZ*	[20]
blaZ2	TACACTCTTGGCGGTTTC			
tetM1	GTTAAATAGTGTTCTTGGAG	656 (45)	*tet*(M)	[21]
tetM2	CTAAGATATGGCTCTAACAA			
tetK1	TTAGGTGAAGGGTTAGGTCC	718 (55)	*tet*(K)	[21]
tetK2	GCAAACTCATTCCAGAAGCA			
aac6F	CAGAGCCTTGGGAAGATGAAG	348 (61)	*aac(6')-Ie-aph(2'')-Ia*	[22]
aac6R	CCTCGTGTAATTCATGTTCTGGC			
aph3F	CCGCTGCGTAAAAGATAC	609 (57)	*aph(3')-IIIa*	[22]
aph3R	GTCATACCACTTGTCCGC			
dftrG1	TTTCTTTGATTGCTGCGATG	501 (51)	*dfr*(G)	[23]
dfrG2	AACGCACCCGTTAACTCAAT			
dfrK1	GCTGCGATGGATAAGAACAG	214 (50)	*dfr*(K)	[24]
dfrK2	GGACGATTTCACAACCATTAAAGC			
ermA1	AAGCGGTAAACCCCTCTGAG	442 (53)	*erm*(A)	[25]
ermA2	TCAAAGCCTGTCGGAATTGG			
ermC1	ATCTTTGAAATCGGCTCAGG	295 (48)	*erm*(C)	[25]
ermC2	CAAACCCGTATTCCACGATT			
msrA1	GCTTAACATGGATGTGG	1230 (55)	*msr*(A)	[22]
msrA2	GATTGTCCTGTTAATTCCC			

Clonality of the isolates was assessed by Pulsed Field Gel Electrophoresis (PFGE) with *Sma*I restriction enzyme according to protocol decribed previously [26]. The definition of a PFGE cluster was based on a similarity cutt-off value of 80% using the unweighted pair group method (UPGMA).

### Antimicrobial susceptibility testing

Antimicrobial susceptibility testing was performed using the broth micro-dilution method. Sensititre® plates and the ARIS 2X automated system (Thermo Scientific) were used with the following antimicrobials: oxacillin, benzylpenicillin, clindamycin, erythromycin, gentamicin, tetracycline, daptomycin, ciprofloxacin, levofloxacin, linezolid, quinupristin/dalfopristin, vancomycin, co-trimoxazole and rifampin. Interpretation of results was carried-out using manufacturers software (SWIN®) adapted to clinical breakpoints of European Committee on antimicrobial susceptibility testing (EUCAST). As there is no clinical breakpoint set for benzylpenicillin for CoNS, the value of 0.12 mg/L designed for *S. aureus* was used. The quality control strain *S. aureus* ATCC 29213 was included in each assay for validation purposes.

### PCR for detection of genes encoding antimicrobial resistance

Detection of genes encoding antimicrobial resistance *(mecA, mecC, blaZ, tet*(K), *tet*(M), *erm*(A), *erm*(C), *msr*(A), *aac(6′)-Ie-aph(2")-Ia, aph(3′)-IIIa*, *dfr*(G) and *dfr*(K) was performed by PCR. Annealing temperatures and oligonucleotides used are presented in Table 1.

### Statistical analysis

Statistical analysis was performed using “R 1.8.1” package (http://www.r-project.org/). Comparison between categorical variables was calculated by chi-square and Fisher’s exact test. Results were considered statistically significant if P < 0.05.

## Results

**Figure 1:**
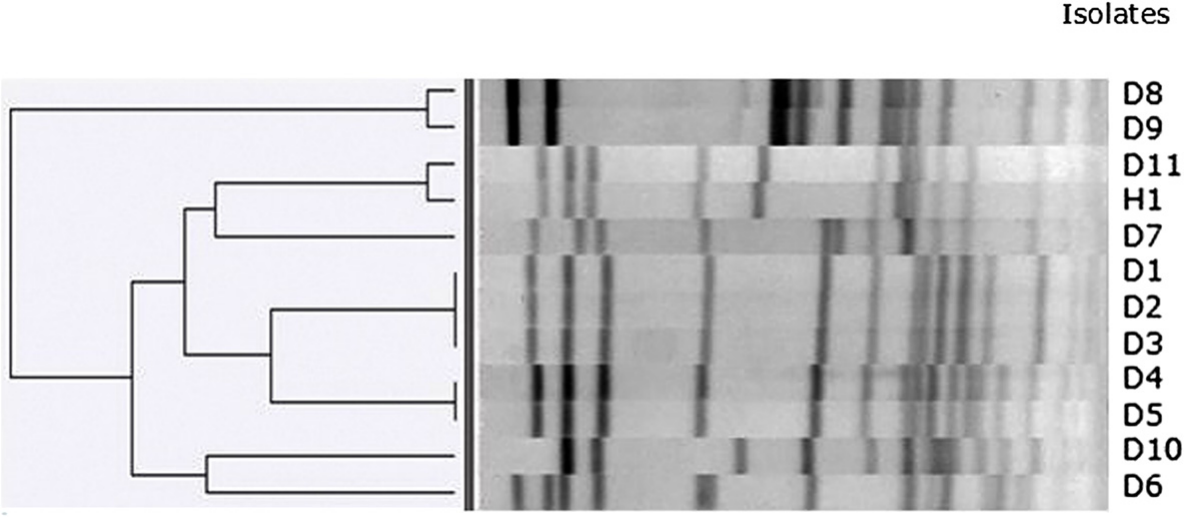
**PFGE dendogram of genomic DNA of MRSH isolates.**

From a total of 754 samples tested, 12 MRSH isolates were obtained (1.6%). No MRSH was found in horses or cats. Eleven isolates were obtained from dogs and one from the dog owner. Ten of the dog isolates were detected in kennels of pure-breed female dogs. Only one MRSH isolate was found in a non-pure-breed 6-year-old dog (male) suffering from deep pyoderma. There were no strains isolated from nose and groin in healthy animals. Three isolates were obtained from the same kennel (breeding English Bulldogs and French Bulldogs). These isolates (D1, D2, and D3) demonstrated the same clonality (Figure 1). They had similar antimicrobial characteristics as well.

One MRSH strain (H1) was obtained from the owner of another kennel (Yorkshire Terriers) and had similar clonality characteristics with one isolate (D11) obtained in this kennel. Two isolates (D4 and D5) from another kennel (breeding English Bulldogs) demonstrated clonality as well (similarity 96%) and had restriction patterns that were close to those of the clone obtained in the first kennel breeding English Bulldogs and French Bulldogs (Figure 1). Those two clones had close antimicrobial susceptibility profiles as well. Small differences were detected in the biochemical properties (reduction of nitrates) among those two clones.

Analysis of the pedigree where two similar MRSH clones were found revealed the existence of the same paternal grandfather among two tested dogs. No other close relations have been registered among those two kennels. The fourth different MRSH clone (isolates D8 and D9, similarity 96%) was detected among two isolates from a kennel breeding German Shepherds.

Statistically reliable results (P < 0.05) for the presence of MRSH were obtained only in the cases of kennels with 6 or more breeding females. Anamnesis data revealed the usage of fluoroquinolones (enrofloxacin) in one MRSH-positive kennel, as well as fluoroquinolones and cephalosporins (cefovecin) in the other kennel during the last three months before sampling. The numbers of female dogs within those kennels were 6 and 18, respectively. The usage of antimicrobials was associated with reproductive disorders in female dogs within those kennels. All MRSH isolates harbored the *mecA* gene, while the *mecC* gene was not detected. Antimicrobial resistance patterns and resistance genes identified in MRSH strains are presented in Table 2.

**Table 2:** **Antimicrobial resistance patterns and resistance genes identified in MRSH strains**

**Isolates**	**Resistance patterns**	**Resistance genes**
D1^a^, D2^a^, D3^a^	OX, P, E, CN, CIP, L, TE, SXT	*mecA, blaZ, tetK*, *ermC, msrA, aac(6′)-Ie-aph(2")-Ia, aph(3′)-IIIa, dfrG*
D4^c^, D5^c^	OX, P, E, CN, CIP, L, TE	*mecA, blaZ, ermA, aac(6′)-Ie-aph(2")-Ia, tetK*
D6	OX, P, E, Q/D	*mecA, blaZ*
D7	OX, CN, TE	*mecA, tetK, aac(6′)-Ie-aph(2")-Ia, aph(3′)-IIIa*
D8^d^, D9^d^	OX, P, E, CLI, CN, SXT	*mecA, blaZ, ermC, msrA, aac(6′)-Ie-aph(2")-Ia, aph(3′)-IIIa, dfrK*
D10	OX, P, E, CLI	*mecA, blaZ, msrA*
D11^b^ H 1^b^,	OX, P, E, CLI, CN, TE	*mecA, blaZ, msrA, tetM, aph(3′)-IIIa*

^a,b,c,d^ – Isolates as the same clone with similar resistance profile; D – dog isolates; H – human isolate.

OX, oxacillin; P, benzylpenicillin; E, erythromycin; CLI, clindamycin; CN, gentamicin; CIP ciprofloxacin; L, levofloxacin; TE, tetracycline; Q/D, quinupristin/dalfopristin; SXT, co-trimoxazole.

**Figure 2:**
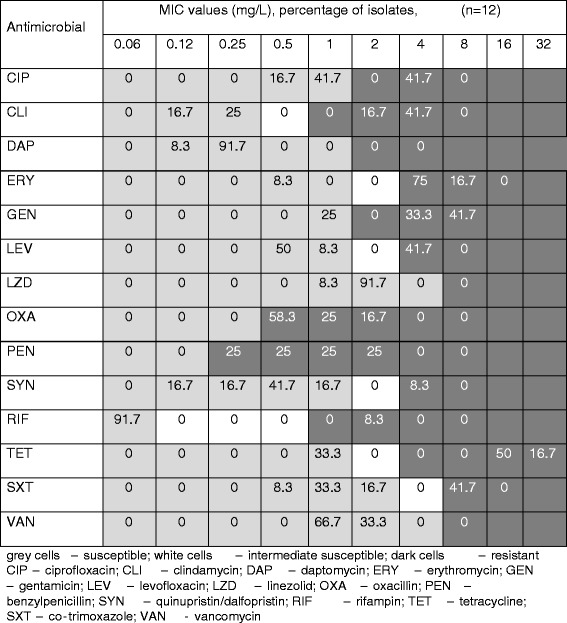
**Minimum inhibitory concentration distributions for the MRSH isolates.**

The most frequent resistances of MRSH isolates demonstrated were to benzylpenicillin (91.7%) with the presence of the *blaZ* gene; erythromycin (91.7%) and clindamycin (41.7%) with the presence of *ermA*, *ermC,* and *msrA* genes; and gentamicin (75.0%) with the presence of *aac(6′)-Ie-aph(2")-Ia* and *aph(3′)-IIIa* genes. Resistance to fluoroquinolones was frequent: 41.7% of the isolates were resistant to ciprofloxacin. *tetK* and/or *tetM* genes were detected in eight tetracycline-resistant isolates (66.7%). Two genes (*dfrG* or *dfrK*) were detected in co-trimoxazole-resistant (41.7%) isolates (Table 1). All isolates were susceptible to daptomycin, rifampin, linezolid, and vancomycin. One isolate was resistant to streptogramins. The clone isolated from a kennel owner and a dog was resistant to beta-lactams, macrolides, gentamicin, and tetracycline. The minimal inhibitory concentrations (MICs) of antimicrobials tested are presented in Figure 2. Statistically reliable correlation between MICs and detected genes was determined for tetracycline (*tet*K gene was associated with MIC16 mg/L), clindamycin (*msr*A gene – 2–4 mg/L), gentamicin (*aph(3′)-IIIa* and *aac(6′)-Ie-aph(2")-Ia* genes – ≥4 mg/L), erythromycin (erm*C* gene – 4 mg/L) and trimethoprim (*dfr*G gene – 8 mg/L).

## Discussion

This study revealed that MRSH is prevalent in the population of dogs, and the occurrence of this species might be associated with specific distribution among certain animal groups. Ten out of twelve MRSH isolates have been detected in female dogs with reproductive disorders in kennels keeping pure-breed dogs. Previous studies did not reveal any specific distribution of this methicillin-resistant species among different animal groups, although separate clones were detected in close-associated sources – mostly animals, the environment, and equipment [12,13]. Reproductive organs of dogs were the main site of MRSH location, whereas no isolates have been found in nasal and groin samples. That might be associated with previous treatment of reproductive infections in bitches, i.e. antimicrobial selective pressure. There are no data about specific anatomical location of *S. haemolyticus* in dogs, although in humans it mostly colonize areas of axillae, perineus and groin [27].

We found MRSH clonality associated with a narrow distribution within separate kennels. The zoonotic character of *S. haemolyticus* is known based on previous facts according its distribution among humans, animals and ready-to-eat meat products [12,28]. Our findings support this, particularly by the fact that the same strain has been found in both dog and dog owner. As a rule, MRSH was detected in large kennels keeping no less than 6 adult female dogs. The data obtained might be important from an epidemiological point of view, as this study revealed a possible risk factor associated with animal concentration and MRS occurrence within the kennels. In spite of the fact that *S. haemolyticus* has previously been found in different animal species in other countries, including horses and cats, we did not find MRSH isolates in either in horses or in cats. This could be associated with the low frequency of methicillin-resistant *Staphylococcus* spp. among animals in Lithuania, as in our previous study, where we found only 4 MRS isolates in 520 livestock samples with no MRS occurrence in horses [29].

MRSH isolates demonstrated resistance to the antimicrobial classes recognized as critically important for humans – fluoroquinolones, macrolides, and aminoglycosides. Previously, intense usage of fluoroquinolones was reported for domestic animal treatment in Lithuania [30,31]. It was also proven that poultry products are frequently contaminated with fluoroquinolone-resistant bacteria [31,32]. Studies in other countries demonstrated frequent acquired resistance of MRSH to fluoroquinolones isolated from humans as well [10,14].

Different genes encoding resistance to antimicrobials have been detected, which supports the opinion that this species could be characterized as highly resistant to antibiotics. The isolates remain susceptible only to vancomycin, linezolid, rifampin, and daptomycin – antibiotics that are not used for animal treatment.

## Conclusions

Dogs were identified as the sole source of MRSH among companion animals in Lithuania. Pure-breed kennels keeping 6 or more females could be recognized as a risk factor for the presence of MRSH strains. MRSH carriers are a potential source for the possible transmission of MRS for their offspring, other animals, and humans in contact. MRSH strains isolated from companion animals were frequently resistant to some classes of critically important antimicrobials, although they remain susceptible to antibiotics exclusively used in human medicine.

## Abbreviations

CoNS: Coagulase-negative staphylococci
MALDI-TOF: Matrix-assisted laser desorption/ionization time-of-flight analysis
MRS: Methicillin-resistant *Staphylococcus*
MRSH: Methicillin-resistant *Staphylococcus haemolyticus*
PCR: Polymerase chain reaction
PFGE: Pulse filed gel electrophoresis
